# The Relationship Between Gambling Problems and the Five-Factor Model of Personality: A Systematic Review and Meta-Analysis

**DOI:** 10.3389/fpsyt.2021.740235

**Published:** 2021-10-12

**Authors:** Rune Strømme, Karine Holthe Børstad, Andrea Eftang Rø, Eilin Kristine Erevik, Dominic Sagoe, Razieh Chegeni, Rune Aune Mentzoni, Puneet Kaur, Ståle Pallesen

**Affiliations:** ^1^Department of Psychosocial Science, University of Bergen, Bergen, Norway; ^2^Norwegian Competence Centre for Gambling and Gaming Research, University of Bergen, Bergen, Norway; ^3^Optentia, The Vaal Triangle Campus of the North-West University, Vanderbijlpark, South Africa

**Keywords:** meta-analysis, personality, problematic gambling, neuroticism, conscientiousness, agreeableness, openness, extroversion

## Abstract

**Objectives:** The aim of the present meta-analysis was to synthesize results from the association between problem gambling (PG) and dimensions of the five factor model of personality and to identify potential moderators (gambling diagnosis: yes/no, comorbidity: yes/no and trait assessment: four or fewer items vs. five items or more) of these associations in meta-regressions.

**Methods:** Searches were conducted in six databases; Medline, Web of Science, PsychInfo, Google Scholar, OpenGrey, and Cochrane Library (conducted on February, 22, 2021). Included studies: (1) reported a relationship between PG and at least one of the personality traits in the five-factor model, (2) contained information of zero-order correlations or sufficient data for such calculations, and (3) were original articles published in any European language. Case-studies, qualitative studies, and reviews were excluded. All articles were independently screened by two authors. Final agreement was reached through discussion or by consulting a third author. Risk of bias of the included studies was assessed by the Newcastle-Ottawa Scale. Data were synthesized using a random effects model.

**Results:** In total 28 studies, comprising 20,587 participants, were included. The correlations between PG and the traits were as follows: Neuroticism: 0.273 (95% *CI* = 0.182, 0.358), conscientiousness −0.296 (95% *CI* = −0.400, −0.185), agreeableness −0.163 (95% *CI* = −0.223, −0.101), openness −0.219 (95% *CI* = −0.308, −0.127), and extroversion −0.083 (*95% CI* = −0.120, −0.046). For all meta-analyses the between study heterogeneity was significant. Presence of gambling diagnosis was the only moderator that significantly explained between-study variance showing a more negative correlation to extroversion when participants had a gambling diagnosis compared to when this was not the case.

**Discussion:** The results indicated some publication bias. Correcting for this by a trim-and-fill procedure showed however that the findings were consistent. Clinicians and researchers should be aware of the associations between personality traits and PG. Previous studies have for example showed neuroticism to be related to treatment relapse, low scores on conscientiousness to predict treatment drop-out and agreeableness to reduce risk of treatment drop-out.

**Systematic Review Registration:** PROSPERO (CRD42021237225).

## Introduction

Gambling can be defined as “*staking possessions of material value on an event with uncertain outcomes(s), that is determined, at least partly, by chance*” [(1), p. 619]. A total of 26% of the population worldwide (amounting to 1.6 billion people) are estimated to gamble regularly (2). For some, gambling turns into a problem or even an addiction, where the severity can be conceptualized along a continuum, ranging from mild problems/few symptoms to more severe cases/many symptoms (3). Prevalence studies indicate that between 0.7 and 6.5% of the adult population in Europe, Asia, North America, and Oceania suffer from problem gambling (PG), i.e., experience some level of harm in relation to their gambling (4). Hence, most gamblers can be regarded as social/recreational gamblers. In terms of those suffering from PG, the subgroup with the most serious problems fulfills the criteria for pathological gambling/gambling disorder (5). Within the latter subgroup one may further differentiate between moderate, severe and extreme cases.

Personality traits, especially traits such as antisocial behavior, impulsivity, and neuroticism can according to temporary models act as risk factors for PG (6, 7). Overall, personality can be defined as relatively stable characteristics of an individual, which affects how one relates to and is influenced by their intrapsychic and social environment (8). Trait theories suggest that personality consists of the combination of several traits or dispositions. The most recognized trait model today is the five-factor model which has emerged as a result of the work of many independent researchers (9, 10). According to this model, personality consists of five relatively stable and unrelated personality dimensions: Neuroticism reflects the presence and effects of negative affect. Accordingly, high scorers on this trait report a range of dysphoric states such as nervous tension, depression, frustration, guilt, and self-consciousness. The second dimension, conscientiousness, has been defined as the will to achieve, and people with high scores on this trait are typically described as thorough, neat, well-organized, diligent, achievement-oriented, and able to hold impulsive behavior in check. The third dimension is agreeableness and involves altruism and trust. High scorers are typically characterized by empathy, nurturance, and caring and as providers of emotional support. The fourth dimension, openness, is associated with some controversy. Some argue that this dimension reflects being intelligent, imaginative, and perceptive, whereas other put more weight on sensitivity to art and beauty when describing the trait. Overall, openness seems to reflect creativity, and intellectual interests, differentiated emotions, aesthetic sensitivity, need for variety, and unconventional values. The last dimension, the extroversion dimension, is rather broad and has been described as being in the midway of dominance and warmth. It reflects characteristics such as being cheerful, enthusiastic, optimistic, energetic, talkative, sociable, and warm (9, 11–13). The five dimensions and being able to detect these dimensions in others, are theorized to have important implications for humans' capabilities of adaptation, survival, and reproduction in evolutionary contexts (14). The five-factor model seems relevant to understand and predict individual differences in how one interacts with different environments, such as gambling environments. A meta-analysis of test-retest correlations of personality throughout life by Roberts and DelVecchio (15), showed that personality traits were relatively stable in early childhood and adolescence, and that they become even more stable in adulthood. These findings which demonstrate that personality traits are likely to be established early in life (i.e., in many cases before the individual starts gambling) suggest that personality may be a precursor for problematic behaviors such as PG. Still, some studies suggest that negative life events can affect and change personality characteristics (e.g., increase neuroticism), which suggest that gambling problems may also lead to personality changes (16).

Several meta-analyses have identified associations between different addictions and the five-factor model. For instance, Kun et al. (17) showed that openness and conscientiousness were positively related to work addiction, whereas Astarini and Yadiarso (18) found that of the five-factor model‘s traits, conscientiousness, and agreeableness had the strongest, and inverse associations with internet addiction. Further, Marengop et al. (19) reported that conscientiousness had the strongest and inverse association with smartphone use disorder and Hakulinen et al. (20) showed that high scores on extroversion and low scores on conscientiousness were associated with heavy alcohol consumption.

In terms of the five-factor model of personality neuroticism is theoretically assumed to be a risk factor for PG (6). Individuals high on neuroticism are assumed to be disposed to impulsivity and emotional vulnerability, which are presupposed to sustain gambling behavior (21). Accordingly, neuroticism has been shown to be positively associated with PG in prevalence studies (22) and has been shown to be related to treatment relapse (23) and to be inversely related to gambling treatment response (24). Regarding conscientiousness an inverse association between this trait and PG is expected as individuals high on conscientiousness are characterized as being tenacious in following long-term personal aims, as well as being structured and organized in their personal issues (25). Excessive gambling can thus be assumed to collide with important long-term personal aims (e.g., happiness and well-functioning social relationships), thus those with high score on conscientiousness may be less likely to engage in excessive gambling. Further, survey studies have shown that those suffering from PG score lower on conscientiousness than their not-suffering counterparts (22). In addition, low scores on conscientiousness have been associated with higher dropout from gambling treatment (23). People high on agreeableness emphasize harmony in interpersonal relationships and can thus be assumed to avoid excessive gambling which often leads to interpersonal conflicts (26). Those with PG has accordingly been shown to have lower scores on agreeableness than non-problems gamblers (22). Further, agreeableness has been shown to be inversely related to gambling treatment attrition (23). When it comes to openness one can assume an inverse association with PG based on the fact that individuals with high scores on openness are known to be highly open-minded to new people and new experiences, in addition to being imaginative, intellectual and creative (12, 27), which are less compatible with PG, which often implies a repetitive behavior toward one activity. Studies have also shown pathological gamblers to score lower than controls on openness (21). It terms of extroversion, we expected there to be a positive association with PG as individuals with high scores on extroversion are more likely to seek out and enjoy external stimulation (26). A study using actual online gambling data attests to this, showing extroversion to be positively associated with online betting intensity (28).

So far, no meta-analysis has been conducted investigating the relationship between the five-factor model and PG. Individual studies do, however, suggest that PG is positively associated with neuroticism and negatively associated with conscientiousness and agreeableness (21, 29, 30). However, the results on the association between PG and extroversion and openness seem more inconsistent (31–35). Against this backdrop, the purpose of the current investigation was to examine the associations between PG and the five-factor model using a meta-analytic approach. Due to partially inconsistent and dissonant findings in previous research on this topic, results of this meta-analysis may pave the way for a better and more comprehensive understanding of the relationship between the five-factor model and PG. Based on theoretical notions and the empirical evidence reviewed above, we postulated five different hypotheses:

H1: There will be a positive association between neuroticism and PG.
H2: There will be an inverse association between conscientiousness and PG.
H3: There will be an inverse association between agreeableness and PG.
H4: There will be an inverse association between openness and PG.
H5: There will be a positive association between extroversion and PG.

## Materials and Methods

The approach and structuring of this meta-analysis were completed according to the Preferred Reporting Items for Systematic Reviews and Meta-analysis (PRISMA) (36). The Appendix in Supplementary Materials shows the PRISMA-check list with references to this paper specifically. This meta-analysis was preregistered at PROSPERO (International Prospective Register of Systematic Reviews; CRD42021237225).

### Eligibility Criteria

Five criteria were used to determine whether a study should be included in the current meta-analysis: (1) The study must report a relationship between PG and at least one of the five personality traits in the five-factor model. PG was defined as any gambling problem ranging from mild gambling problems to gambling disorder, either diagnosed clinically or assessed by any instrument assessing gambling problems. (2) The personality traits of the five-factor model had to be assessed with any relevant self- or observer-rating instrument or item. (3) If the data involved group comparisons the comparator comprised a non-gambling problem group. (4) The included studies must have reported the zero-order correlations between PG and personality traits, or sufficient data for such calculations, such as *N*, mean scores, and standard deviations for the PG and a comparison group. (5) The studies must have reported original data. We included studies written in all European languages. There were no criteria concerning population, interventions, participant characteristics, or year of publication. Studies were excluded if they were: (1) based on case-studies, (2) were qualitative studies, (3) were reviews or only reported secondary data, or (4) measuring personality traits similar to the five-factor model, but not the actual traits.

### Search Strategy and Data Extraction

**Figure 1:**
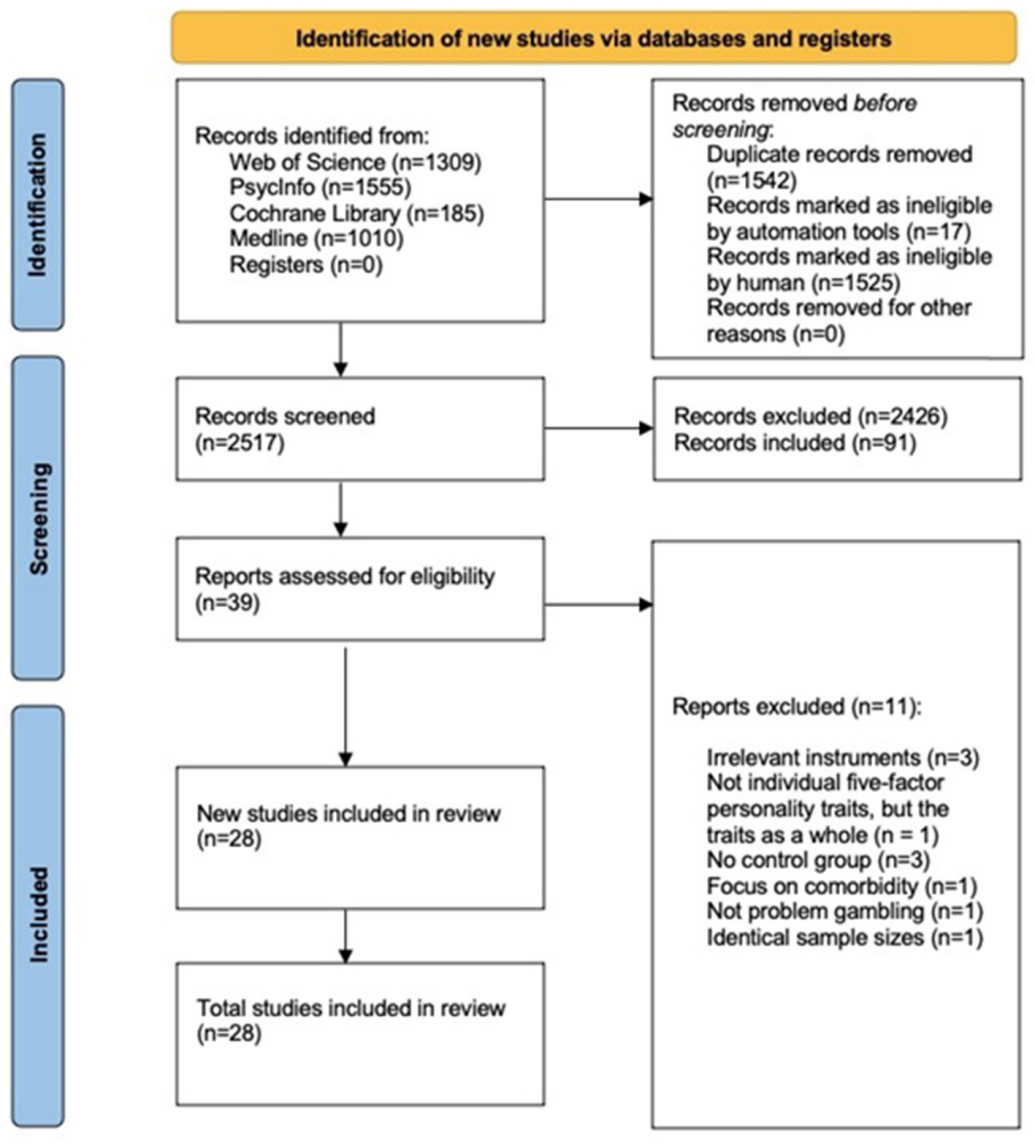
Flow chart depicting the search and inclusion process.

Relevant publications were collected through a search of six research databases: Medline, Web of Science, PsychInfo, Cochrane Library, OpenGrey, and Google Scholar. The following search string was designed to locate the articles of interest and their relevance to the meta-analysis: “(gambl^*^) AND (personality OR “five factor” OR neuroticism OR extraversion OR extroversion OR openness OR intellect OR agreeableness OR conscientiousness).” The searches in each database ended on February 2nd, 2021. Based on this search string we identified a total of 4,059 articles of interest. The number of articles identified from each database were: Web of Science: 1,309, PsycInfo: 1,555, Cochrane: 185, and Medline: 1,010. The searches in Google Scholar and OpenGrey were conducted to identify gray literature, but no additional literature was identified through these sources. After removing duplicates, 2,517 articles were available for screening. Of these, 2,427 were removed as they were deemed irrelevant based on title and abstract. After screening the remaining 91 full-text articles, 45 articles were included for detailed screening. In all, 17 articles [e.g., (37, 38)] were excluded as they did not fulfill the inclusion criteria (see Figure 1). Thus, a total of 28 articles were included in the present meta- analysis.

To ensure validity, as well as avoid mistakes regarding adherence to the inclusion and exclusion criteria, all articles were systematically and independently reviewed by two authors. Observer agreement was expressed as percentages. Disagreements were resolved by discussion, and if needed by consulting a third team member. Data from the selected studies (zero-order correlations between PG and the personality factors and information regarding possible moderators) were coded separately by two authors. Disagreements were resolved by discussions. For studies with appropriate data from more than one personality instrument, or in studies reporting data from at least two subdimensions of each dimension of the five-factor model, the data were entered combined.

### Risk of Bias Assessment

The studies included in the meta-analysis were assessed by the Newcastle-Ottawa Scale for cross-sectional studies (39) to identify risk of bias/study quality. The Newcastle-Ottawa Scale provides predefined criteria for assessing bias/quality through a checklist, consisting of three main categories. It is possible to score a maximum of 10 stars, where a higher score indicates higher quality/less bias. The first category is “selection,” and relates to the representativeness of the sample, sample size, comparability between respondents and non-respondents, and ascertainment of the exposure. This category gives maximum of five stars. The second category is “comparability,” and concerns whether confounding factors are controlled for. This category gives maximum two stars. The third category is “outcome” and represents assessment of the outcome and the statistical tests. The maximum score on this category is three stars. In the current study, studies with five stars or more were considered to have moderate to good quality. Two authors evaluated the papers for risk of bias independently. Disagreements were resolved by discussion. Degree of agreement was calculated as percentages.

### Statistical Analysis

We used a random-effects model in the meta-analysis of correlations between PG and personality. A random-effects model was chosen as this approach is likely to improve the external validity and generalizability of findings and is recommended when included studies are assumed to represent different populations of studies (40). Results from individual studies and syntheses were visualized as forest plots including single and accumulated effect sizes (correlation) as well as 95% confidence intervals. In order to explore factors that might explain between-study heterogeneity, we conducted a random-effects meta-regression analysis to examine whether three a priori determined moderators could explain between-study variance. The first moderator comprised level of PG. This involved whether the sample was based on participants with a diagnosed gambling disorder (coded as 1) or participants assumingly having PG, albeit without a formal diagnosis (coded as 0). The second moderator concerned comorbidity. If more than 50% of the participants had a comorbid disorder this was coded as 1, if 50% or more did not have a comorbid disorder this was coded as 0. The last moderator comprised assessment of personality. If the scale had four items or fewer this was coded as 0, and if the scale had five items or more this was coded as 1. Heterogeneity was assessed using Cochrans' *Q*. The *I*^2^ statistic, which reflects the proportion of variation in observed effects that is due to variation in true effects (i.e., “true” heterogeneity as opposed to chance) (40), was also calculated. An *I*^2^ of 0% suggests homogeneity, 25% indicates low heterogeneity, 50% indicates moderate heterogeneity, and 75% indicates high heterogeneity (41). The trim-and-fill procedure by Duval and Tweedie (42) was used for investigation of publication bias. The meta-analysis and meta-regression analyses were conducted using the Comprehensive Meta-Analysis 3.0 software (43). The observer agreement on data-coding of effect sizes, moderators and quality assessment were calculated percentwise.

## Results

### Description of Included Studies

The included studies were collected from 13 countries, between the years 1987 to 2020. Sample sizes in the included studies varied from 37 to 9,111. The instruments used to measure PG were, in most of the studies, the South Oaks Gambling Screen [SOGS; (44)], with a close follow up by instruments based on the criteria found in third, fourth, and fifth editions of the Diagnostic and Statistical Manual of Mental Disorders [DSM-III, DSM-IV, DSM-5; (45–47)]. Other instruments were the Problem Gambling Severity Index/Canadian Problem Gambling Index [PGSI/CPGI; (48)], the Lie/bet Questionnaire [LBQ; (49)], the Shorter PROMIS Questionnaire [SPQ; (50)], the Structured Clinical Interview for Pathological Gambling [SCI-PG; (51)], the International Classification of Disorders-10 [ICD-10; (52)], the Berlin Inventory of Gambling [BIG; (53)], the National Opinion Research Center DSM-IV Screen for Gambling Problems [NODS; (54)], the Canadian Adolescent Gambling Inventory [CAGI; (55)], the DSM-IV-Multiple Response-Juvenile Criteria to Identify Adolescent Problem Gambling [DSM-IV-MR-J; (56)], and the Gambling Problems Index [GPI; (57)]. The most common measurement tool for personality was the Revised NEO Personality Inventory Revised [NEO-PI-R; (58)], which was used in seven studies. Other measurement tools for personality were the Eysenck Personality Inventory [EPI; (59)], the Eysenck Personality Questionnaire [EPQ; (60)], the Mini-International Personality Item Pool [MINI-IPIP; (61)], the Big Five Inventory [BFI; (62)], the 16 Personality Factor Questionnaire [16PF; (63)], the International Personality Item Pool NEO 120 [IPIP NEO 120; (64)], the Estonian Personality Item Pool-NEO [EPIP-NEO; (65)], the HEXACO Personality Inventory Revised, HEXACO-60 [HEXACO-60; (66)], the NEO-Five Factor Inventory [NEO-FFI; (67)], the HEXACO-Personality-Inventory-Revised [HEXACO PI-R; (68)], the Zuckerman-Kuhlman Personality Questionnaire [ZKPQ; (69)], and the Brief HEXACO Inventory [BHI; (70)].

The number of personality dimensions of the five-factor model that each study measured varied from one to five. A summary of the characteristics of each study, including country, publication, sample sizes of PG, and control group, instrument used for assessment of personality and gambling problems, number of personality dimensions measured, and the measure of effect size, is provided in Table 1. The agreement between the independent observers regarding coding of these characteristics were 98.1%. The agreement of coding of correlation data was calculated as percentages and amounted to 75.6%. The moderators used in the meta-regression analyses were coded independently by two authors and agreement was 62.5% for diagnosis 83.3% for comorbidity and 75.0% for number of personality items, respectively.

**Table 1:** Characteristics of study articles.

**References**	**Country**	**Publication type**	**Sample size pathological gamblers**	**Sample size control group**	**Gender**	**Age**	**Study goal**	**Diagnostic method**	**Measuring instrument gambling problems**	**Measuring instrument personality dimensions**	**No. of personality dimensions of the five-factor model measured**	**Effect size based on**
(29)	Canada	Journal	106	177	146 F; 137 M	N/A	Personality differences between non-treatment seeking PG's and NPG's	N/A	DSM-IV	NEO PI-R	5	Mean/Standard deviation
(71)	India	Journal	20	20	40 M	25–50	CG indulge in gambling activities to overcome personal inadequacies	N/A	Known compulsive gamblers	EPI	2	Mean/Standard deviation
(72)	Australia	Journal	115	404	14 F; 505 M	17–73	The role of impulsivity in PG's	Semi-structured interview; psychometric tests	DSM-III	EPQ	2	Mean/Standard deviation
(22)	Norway	Journal	57	9,054	4,282 F;4,829 M	Mean age 47	Differences in neuroticism, extroversion, intellect, agreeableness, and conscientiousness between NPG's and low-, moderate-, and severe PG's	Questionnaire by postal mail	PGSI	MINI-IPIP	5	Mean/Standard deviation
(73)	Italy	Journal	30	80	54 F; 56 M	Mean age 37	Relationship of GD risk to adaptive and maladaptive personality dimensions	Self-report questionnaire	LBQ	BFI	5	Mean/Standard deviation
(74)	Italy	Journal	40	160	13 F; 187 M	30–50.5	Study of classification algorithms ability to discriminate individuals with GD from C	Questionnaires administered by at least one clinical research trained psychologist	DSM-5	NEO PI-R	5	Mean/Standard deviation
(31)	Australia	Journal	15	34	29 F; 20 M	18–38	Relationship between individual factors and levels of gambling involvement	Questionnaires completed in groups of 1–10 participants in presence of an administrator	CPGI	16PF	1	Mean/standard deviation
(75)	USA	Journal	812	N/A	N/A	N/A	Effect of positive expectancies on coping motives in development of PG	Online survey	SOGS	IPIP-NEO-120	1	Pearson's correlation
(32)	Republic of Korea	Journal	15	33	48 M	Mean age PG 28.2; C 34.9	Explore whether PG resembles obsessive-compulsive disorder in terms of personality and temperament	Clinical assessment	SOGS	NEO PI-R	5	Mean/standard deviation
(76)	Estonia	Journal	32	37	N/A	N/A	Identify psychological characteristics of Estonian PG's	Structured clinical interview	SOGS	EPIP-NEO	5	Mean/Standard deviation
(77)	USA	Journal	326	N/A	156 F; 170 M	Mean age G 33.2; NG 36.1	Assessing whether the HEXACO dimensions are associated with both current gambling status and gambling severity	N/A	PGSI	HEXACO	5	Pearson's correlation
(78)	Canada	Journal	369	N/A	284 F; 85 M	18–25	Correlation between personality and problem gamblers	Self-report in anonymous group testing	SPQ	NEO PI-R	5	Pearson's correlation
(79)	Canada	Journal	273	N/A	146 F; 123 M	18–63	Examine gambling motives, distorted beliefs about gambling, and personality traits	Self-report in anonymous group testing	PGSI	NEO PI-R	4	Pearson's correlation
(80)	Germany	Journal	515	269	N/A	Mean age PG 38; C 35	Prevalence of comorbidity, family history, and personality traits in PG's	Clinical records	ICD-10	NEO-FFI	5	Mean/Standard deviation
(33)	Canada	Journal	326	N/A	193 F; 133 M	Mean age 21	Assessing whether the HEXACO dimensions are associated with gambling engagement and gambling severity	Self- and observer reports	PGSI	HEXACO-PI-R	5	Pearson's correlation
(34)	USA	Journal	354	N/A	78 F; 276 M	18–64	Relations of PG; and big three and FFM	Diagnostic interview and self-report	SCI-PG	NEO-FFI/BFI	5	Pearson's correlation
(81)	Hong Kong	Dissertation	70	45	31 F; 84 M	18–60 or above	Demographic and personality factors as predictors of PG	Self-administered questionnaire	SOGS	ZKPQ	1	Mean/standard deviation
(82)	Canada	Journal	327	N/A	N/A	N/A	Examine if protective factors explain variance in problem gambling tendencies beyond the HEXACO personality traits	Self-report	PGSI	BHI	5	Pearson's correlation
(25)	Germany	Journal	122	93	N/A	Mean age PG 32.3; C 21	Evaluate the relationship between personality traits and IGD	Self-report	BIG	NEO-FFI	5	Mean/Standard deviation
(21)	Norway	Journal	90	66	47 F; 109 M	Mean age PG 37.9; C 40.2	Investigate the relationship between different personality variables and PG	N/A	SOGS-R	NEO-FFI	5	Mean/Standard deviation
(83)	Spain	Journal	44	88	12 F; 120 M	21–75	Assess personality profile and predict treatment outcome of treatment-seeking adult outpatients with PG	N/A	NODS	ZKPQ	1	Mean/Standard deviation
(84)	USA	Journal	226	N/A	116 F; 110 M	13–17	Investigate whether sensation-seeking better predicts gambling behavior than personality	Online survey self-report	CAGI/DSM-IV-MR-J	BFI	5	Pearson's correlation
(85)	USA	Journal	69	55	33 F; 91 M	18–82	Exploring facets of personality and escapism in PG's	Self-report	SOGS-R	NEO-PI-R	5	Mean/Standard deviation
(86)	USA	Journal	19	18	37 M	N/A	Difference in personality factors in PG's and NPG's	Competing personality questionnaires	DSM-III	EPQ	2	Mean/Standard deviation
(30)	Switzerland	Journal	4,989	N/A	4,989 M	Mean age 21.26	PGA difference in GP, substance use outcomes, personality traits, and coping strategies	Self-report	DSM-5	ZKPQ	1	Mean/Standard deviation
(87)	USA	Journal	220	N/A	91 F; 129 M	18–52	Associations among FFM, motives for gambling and gambling behavior	Online self-report	NODS/ SOGS/ GPI	BFI	5	Pearson's correlation
(35)		Journal	103	145	86 F; 162 M	N/A	Relationships among FFM personality measures and forms of gambling in adults with or without probable PG	Questionnaire	SOGS	NEO-PI-R	5	Mean/Standard deviation
(88)	Israel	Journal	47	78	57 F; 67 M	N/A	Personality profiles of substance and behavioral addictions	Online questionnaire	SOGS	BFI	5	Mean/Standard deviation

*SOGS, South Oaks Gambling Screen; DSM-IV, DSM-III, and DSM-5, fourth, third, and fifth version of the Diagnostic and Statistical Manual of Mental Disorders; PGSI/CPGI, Problem Gambling Severity Index/Canadian Problem Gambling Index; LBQ, the Lie/bet Questionnaire; SPQ, Shorter Promise Questionnaire; SCI-PG, Structured Clinical Interview for Pathological Gambling; ICD-10, International Classification of Disorders-10; BIG, Berlin Inventory of Gambling; NODS, National Opinion Research Center DSM-IV Screen for Gambling Problems (NODS, 54); CAGI, Canadian Adolescent Gambling Inventory; DSM-IV-MR-J, DSM-IV-Multiple Response-Juvenile Criteria to Identify Adolescent Problem Gambling; GPI, Gambling Problems Index; NEO-PI-R, Revised NEO Personality Inventory Revised; EPI, Eysenck Personality Inventory; EPQ, Eysenck Personality Questionnaire; MINI-IPIP, Mini-International Personality Item Pool; BFI, Big Five Inventory; 16PF, 16 Personality Factor Questionnaire; IPIP NEO 120, International Personality Item Pool NEO 120; EPIP-NEO, Estonian Personality Item Pool-NEO; HEXACO-60, HEXACO Personality Inventory Revised; NEO-FFI, NEO-Five Factor Inventory; HEXAO-PI-R, HEXACO-Personality-Inventory-Revised; ZKPQ, Zuckerman-Kuhlman Personality Questionnaire; BHI, Brief HEXACO Inventory; F, Female; M, Male; G, Gamblers; PG, Pathological Gamblers; NG, Non-Gamblers; NPG, Non-Pathological Gamblers; C, Controls; CG, Compulsive Gamblers; GD, Gambling Disorder; FFM, Five Factor Model of personality; IGD, Internet Gaming Disorder; PGA, Patterns of Gambling Activities*.

### Risk of Bias of the Included Studies

The Newcastle-Ottawa Score results for each study are shown in Table 2. Five of the included studies obtained less than five stars and were therefore considered to be of low quality. The remaining 23 studies obtained five or more stars and were therefore considered to be of moderate or high quality. The average quality score was 5.89 (*SD* = 1.69). The agreement between raters were 78.6% for “selection,” 100.0% for “comparability,” and 96.4% for “outcome,” respectively.

**Table 2:** The results of quality/risk of bias assessment based on the Newcastle-Ottawa Scale.

**References**	**Selection**	**Comparability**	**Outcome**	**Total**
(29)	***		*	****
(71)	**			**
(72)	***	**	*	******
(22)	*****	**	**	*********
(73)	****		*	*****
(74)	**	**	*	*****
(31)	***	**	*	******
(75)	****	**	*	*******
(32)	**	**		****
(76)	***	**	**	*******
(77)	****	**	**	********
(78)	***		*	****
(80)	****	**	*	*******
(33)	***	**	**	*******
(34)	*****		*	******
(81)	***	**	*	******
(82)	***	**	*	******
(21)	****	**	**	********
(83)	***		**	*****
(84)	****	**	*	*******
(85)	****		*	*****
(86)	*		*	**
(30)	****	**	*	*******
(87)	****	**	**	********
(35)	***	**	**	*******
(25)	****	*	*	******
(79)	***		**	*****
(88)	***	**	*	******

### Results of Individual Studies and Syntheses

**Figure 2:**
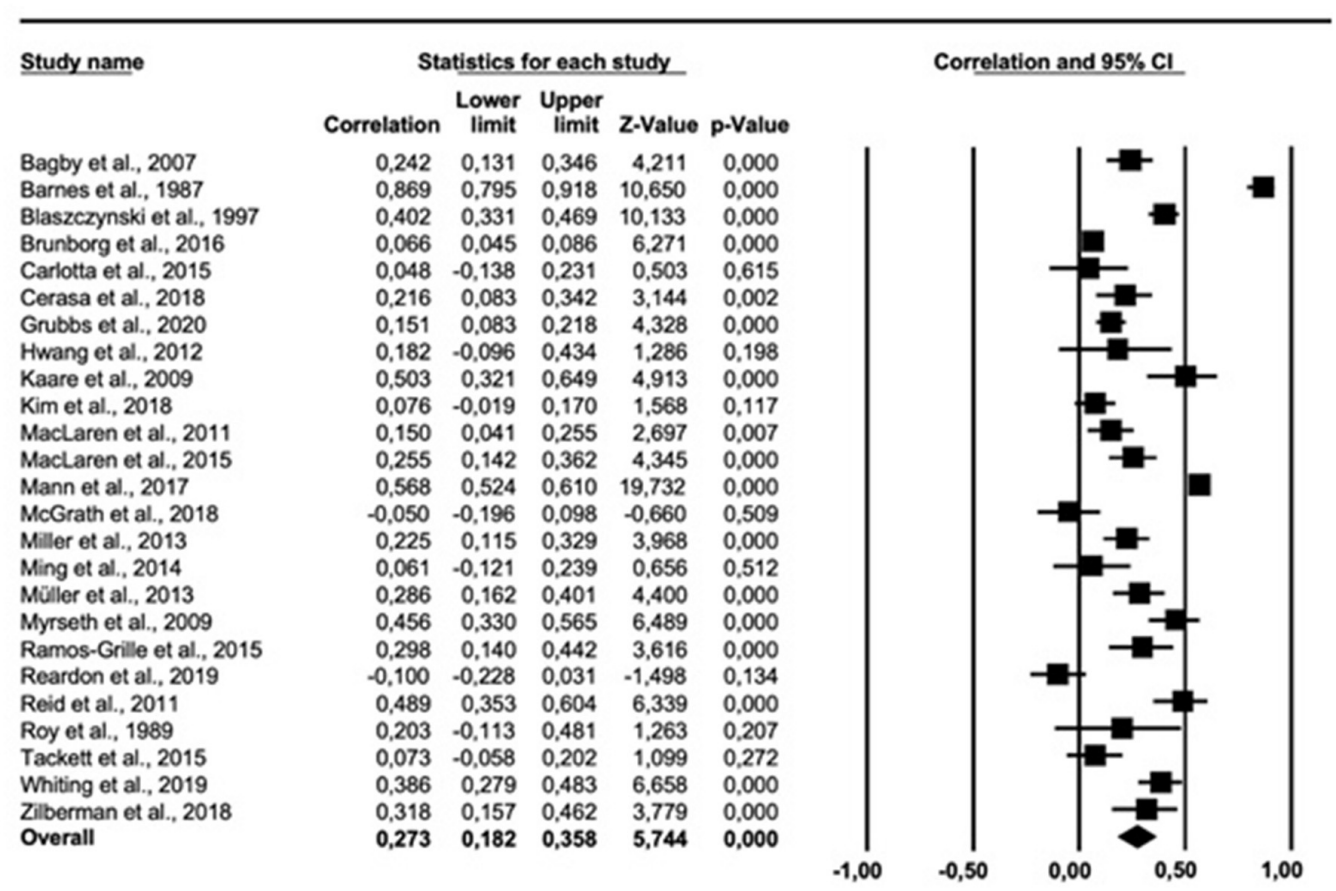
Forest plot showing the relationship between gambling problems and neuroticism.

The results of the meta-analysis on the personality trait neuroticism are presented in Figure 2. The overall correlation across 25 relevant studies was 0.273 (95% *CI* = 0.182, 0.358). Cochran *Q* was significant (*Q* = 544.801, *df* = 24, *p* < 0.001). The *I*^2^ was 95.78% indicating a high heterogeneity. The funnel plot (Appendix in Supplementary Materials) suggested an asymmetric distribution of correlations. The trim-fill-prodedure suggested an adjusted effect size of 0.378 (95% *CI* = 0.258, 0.486).

**Figure 3:**
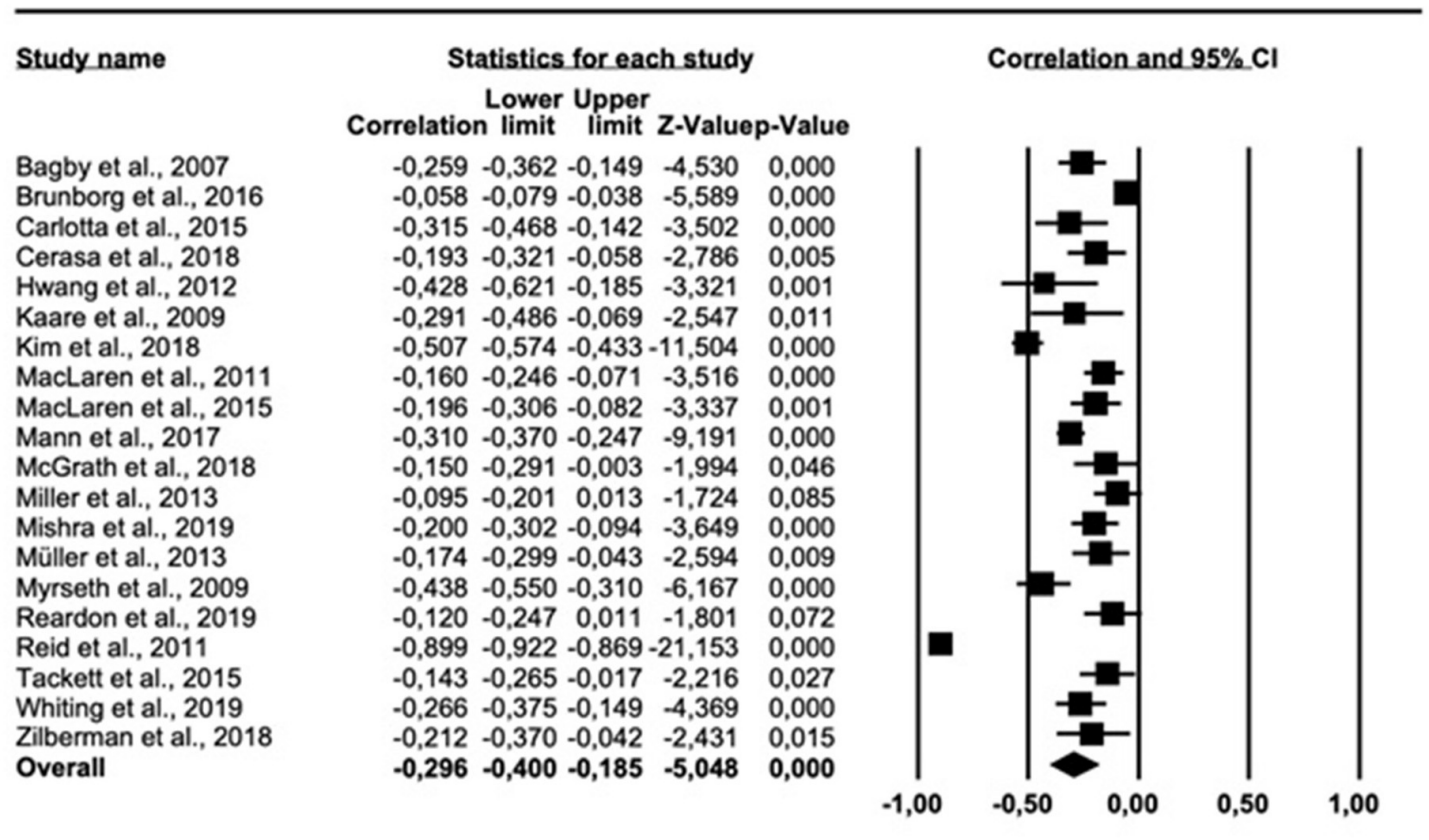
Forest plot showing the relationship between gambling problems and conscientiousness.

The results of the meta-analysis on the personality trait conscientiousness are shown in Figure 3. The overall correlation across all 20 relevant study articles was −0.296 (95% *CI* = −0.400, −0.185). Cochran *Q* was significant (*Q* = 570.959, *df* = 19, *p* < 0.001), suggesting significant heterogeneity across studies. The *I*^2^ was 96.670%, indicating a high heterogeneity. The funnel plot (Appendix in Supplementary Materials) suggested an asymmetric distribution of correlations. The trim-fill-prodedure suggested an adjusted effect size −0.398 (95% *CI* = −0.517, −0.264).

**Figure 4:**
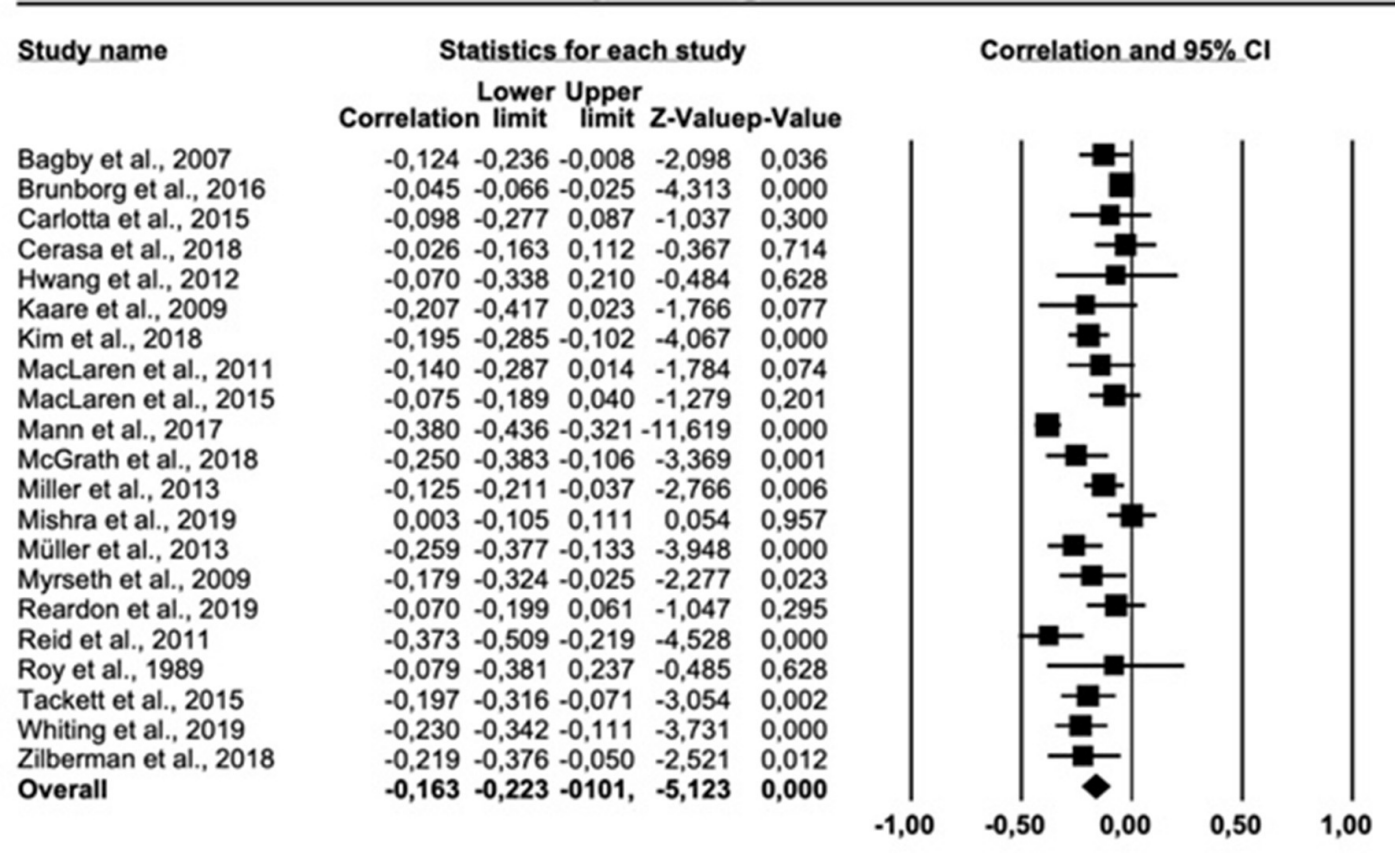
Forest plot showing the relationship between gambling problems and agreeableness.

The result of the meta-analysis on the personality trait agreeableness are presented in Figure 4. The overall correlation across all 21 relevant studies was −0.163 (95% *CI* = −0.223, −0.101). Cochrans' *Q* was significant (*Q* = 145.237, *df* = 20, *p* < 0.001), suggesting heterogeneity. The *I*^2^ was 86.229%, indicating high heterogeneity. The funnel plot (Appendix in Supplementary Materials) suggested an asymmeteic distribution of correlations. The trim-fill-prodedure suggested an adjusted effect size −0.193 (95% *CI* =-0.264, −0.121).

**Figure 5:**
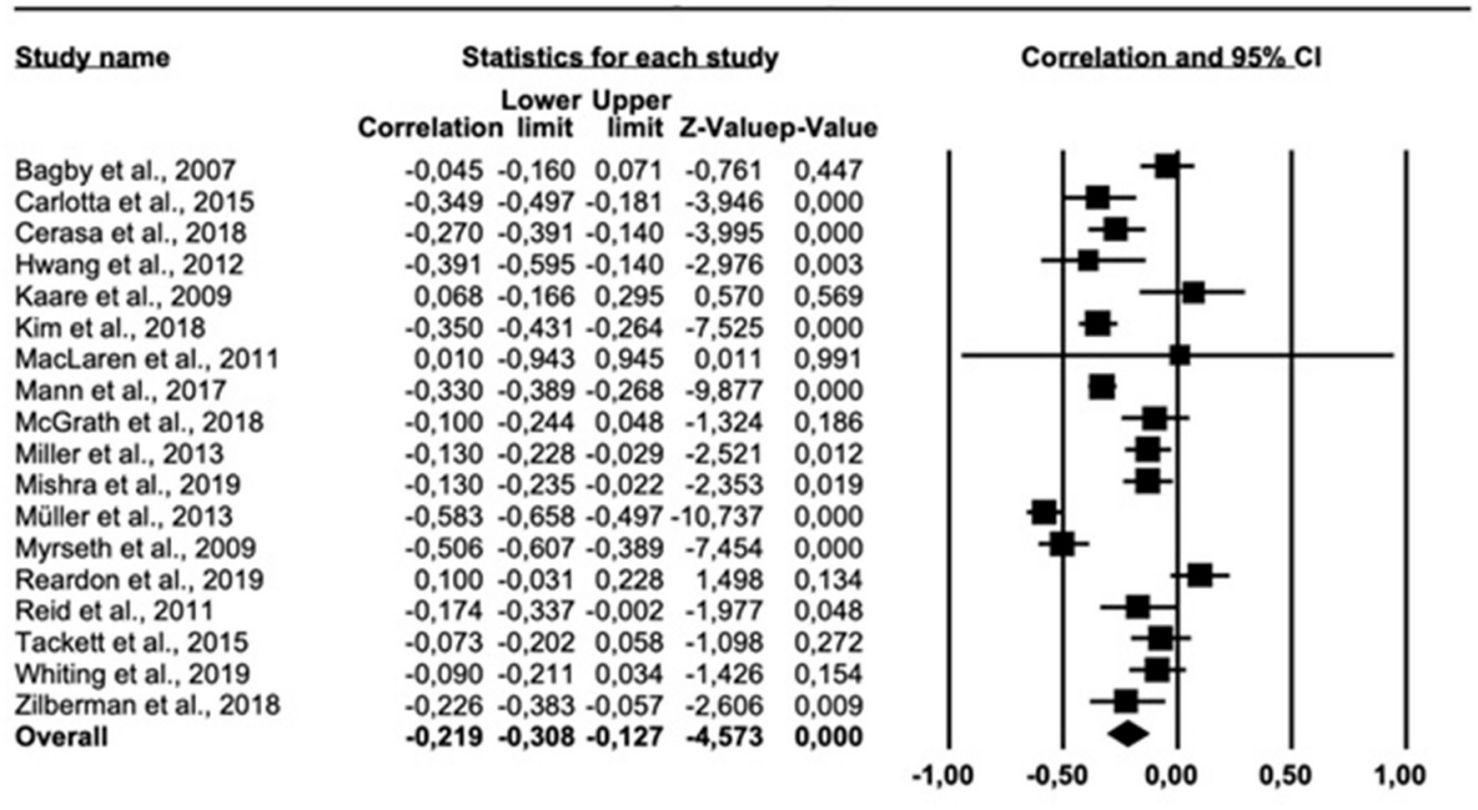
Forest plot showing the relationship between gambling problems and openness to experience.

The results of the meta-analysis on the personality trait openness are presented in Figure 5. The overall correlation across all 18 relevant studies was −0.219 (95% *CI* = −0.308, −0.127). Cochran *Q* was significant (*Q* = 151.651, *df* = 17, *p* < 0.001), suggesting significant heterogeneity. The *I*^2^ statistic was 88.790%, indicating a high heterogeneity. The funnel plot (Appendix in Supplementary Materials) suggested an asymmetric distribution of correlations. The trim-fill-prodedure suggested an adjusted effect size of −0.256 (95% *CI* = −0.344, −0.163).

**Figure 6:**
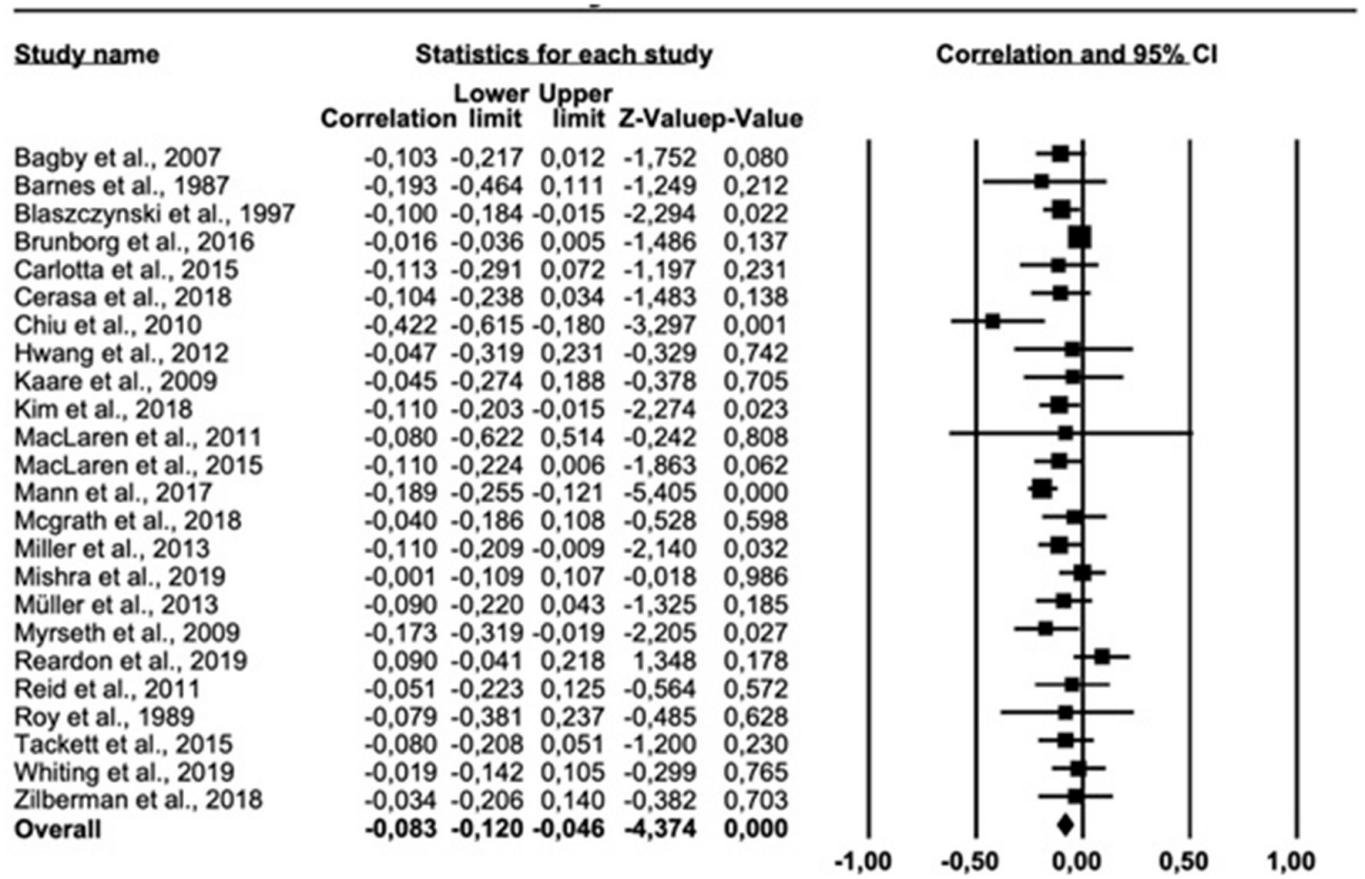
Forest plot showing the relationship between gambling problems and extroversion.

The results of the meta-analysis on the personality trait extroversion are presented in Figure 6. The overall correlation across all 24 relevant studies was −0.083 (95% *CI* = −0.120, −0.046). Cochran *Q* was significant (*Q* = 51.538, *df* = 23, *p* < 0.005), suggesting heterogeneity. The *I*^2^ was 55.373%, indicating a moderate heterogeneity. The funnel plot (Appendix in Supplementary Materials) suggested a symmetric distribution of correlations. The trim-fill-prodedure did not change the outcome for extroversion.

### Predictors of Between Study Variances

Because of the significant heterogeneity in all meta-analyses, a meta-regression analysis based on a random-effect model was conducted for each trait. The results are presented in Table 3. For the personality dimensions including neuroticism (*Q* = 2.62, *df* = 3, *p* = 0.455, *R*^2^ = 22%), conscientiousness (*Q* = 0.62, *df* = 3, *p* = 0.893, *R*^2^ = 0%), agreeableness (*Q* = 1.06, *df* = 3, *p* = 0.788, *R*^2^ = 36%), and openness (*Q* = 5.0, *df* = 3, *p* = 0.170, *R*^2^ = 8%) the regression model was not significant. The regression model was however significant for extroversion (*Q* = 7.91, *df* = 3, *p* = 0.048, *R*^2^ = 73%). Only the predictor, diagnosis, was significant (*b* = −0.0797, *p* = 0.029), suggesting more negative effect sizes for samples with participants with a gambling diagnosis than for samples without a gambling diagnosis. There was still significant unexplained between-study variance for extroversion (*Q* = 51.54, *df* = 23, *p* = 0.001).

**Table 3:** Meta-regression analysis summary for diagnosis, comorbidity, and number of personality items predicting the overall correlation effect size.

**Predictor**	**Coefficient**	**Standard error**	**95% CI**	**Z-value**	**Two-sided *P*-value**
**Neuroticism** (*k* = 24, *R^2^* = 0.22)					
Intercept	0.312	0.099	0.118, 0.507	3.15	0.002
Diagnoses (No = 0, Yes = 1)	0.163	0.109	−0.050, 0.376	1.50	0.134
Comorbidity (No = 0, Yes = 1)	−0.05	0.113	−0.272, 0.170	−0.45	0.652
Number of personality items (<4 = 0, >5 = 1)	−0.112	0.118	−0.343, 0.119	−0.95	0.341
**Conscientiousness** (*k* = 20, *R^2^* = 0.00)					
Intercept	−0.175	0.214	−0.594, 0.243	−0.82	0.412
Diagnoses (No = 0, Yes = 1)	0.017	0.161	−0.298, 0.331	0.10	0.918
Comorbidity (No = 0, Yes = 1)	0.062	0.179	−0.289, 0.412	0.35	0.730
Number of personality items (<4 = 0, >5 = 1)	−0.169	0.235	−0.628, 0.290	−0.72	0.471
**Agreeableness** (*k* = 18, *R^2^* = 0.36)					
Intercept	−0.159	0.086	−0.327, 0.010	−1.84	0.065
Diagnoses (No = 0, Yes = 1)	−0.045	0.061	−0.165, 0.075	−0.74	0.461
Comorbidity (No = 0, Yes = 1)	−0.029	0.066	−0.158, 0.100	−0.44	0.660
Number of personality items (<4 = 0, >5 = 1)	0.022	0.095	−0.169, 0.207	0.23	0.820
**Openness** (*k* = 18, *R^2^* = 0.08)					
Intercept	−0.100	0.194	−0.481, 0.280	−0.52	0.606
Diagnoses (No = 0, Yes = 1)	−0.147	0.098	−0.338, 0.045	−1.50	0.134
Comorbidity (No = 0, Yes = 1)	−0.131	0.099	−0.324, 0.063	−1.32	0.186
Number of personality items (<4 = 0, >5 = 1)	−0.003	0.209	−0.412, 0.406	−0.01	0.989
**Extroversion** (*k* = 24, *R^2^* = 0.73)					
Intercept	−0.117	0.042	−0.199, −0.034	−2.77	0.006
Diagnoses (No = 0, Yes = 1)	−0.080	0.036	−0.151, −0.008	−2.19	0.029
Comorbidity (No = 0, Yes = 1)	−0.008	0.038	−0.082, 0.067	−0.20	0.845
Number of personality items (<4 = 0, >5 = 1)	0.076	0.047	−0.015, 0.167	1.63	0.103

*k, number of studies*.

## Discussion

This quantitative review examined the correlation between PG and the dimensions of the five-factor model of personality. A total of 28 studies fulfilled the inclusion criteria. Four of the five postulated hypotheses were supported. All five meta-analyses turned out significant. The two strongest (*|r|* > 0.25) correlations were found for conscientiousness (*r* = − 0.296) and neuroticism (*r* = 0.273) whereas the smallest correlation (*|r|* < 0.10) was found for extroversion (*r* = 0.083). The results for each dimension are further discussed in detail below.

H1 (expecting a positive association between PG and neuroticism) was supported with an overall *r* of 0.273. It is reasonable to assume that the relationship between PG and neuroticism is bidirectional by nature (34). Gambling may act as a distraction from anxiety and difficulties in life (6, 21). The view that gambling may function as an escape from dysphoric feelings is further compatible with the assumptions of both Roberts and DelVecchio (15) and Specht et al. (16). In terms of possible reverse causation, it has been argued that financial and interpersonal consequences of PG can produce an increase in anger and guilt and as such contribute to increased scores on neuroticism (21).

H2 (expecting an inverse association between PG and conscientiousness) was supported with an overall correlation of −0.296. Müller et al. (25) indicated that individuals low on conscientiousness are characterized by being less tenacious in following personal aims, as well as being unstructured and disorganized in their personal issues. Brunborg et al. (22) further described problem gamblers as individuals with an incapability to think through long-term consequences. The observed inverse association between PG and conscientiousness may thus reflect that individuals with low scores on conscientiousness have trouble seeing and correcting their behavior in accordance with the negative consequences of their gambling behavior. In addition to low conscientiousness scores possibly leading to PG, it is also possible that the association may, in part, be explained by PG causing a decrease in conscientiousness or common third variables causing both PG and lower conscientiousness scores. In line with the latter, some studies have found that risky behaviors (e.g., substance use) can predict a decrease in conscientiousness scores (89, 90). Further, socioeconomic status is an example of a characteristic that may act as a third variable in the relationship between PG and conscientiousness, as socioeconomic status is known to predict both PG and conscientiousness (91, 92).

The results further supported H3 (expecting an inverse association between PG and agreeableness), with an overall correlation of −0.163. Individuals low on agreeableness are often characterized by being competitive, challenging, and less cooperative with others (25). Gambling itself is often characterized by competition suggesting that low scores on agreeableness can be expected to be quite frequent among problem gamblers (25). Another line of reasoning relates to interpersonal functioning. As individuals with high scores on agreeableness typically are motivated to avoid interpersonal conflicts, which often are a result of PG, it seems reasonable to expect agreeableness to be a protective factor for the development of gambling problems (22, 26). It is further possible that the relationship between PG and agreeableness is bidirectional. PG could be speculated to lower agreeableness, as such problems may increase focus on self and lessen the focus on other people.

H4 (expecting an inverse association between GP and openness) was also supported with an overall correlation of −0.219. In general, individuals with a high score on openness are more open-minded to new people and new experiences, in addition to being imaginative, intellectual, and creative (27). According to Müller et al. (25) problem gamblers tend to stay in comfortable and well-known environments related to gambling, instead of seeking out new settings and explore novel environments. These notions are in line with the current finding of an inverse correlation between openness and PG. Another possible explanation for this finding is that individuals high on openness are considered to be concerned with intellectual processes, and may acknowledge the negative outcomes of gambling, and thus be more likely to avoid gambling compared to those with low scores on openness (12). The association may also in part be explained by socioeconomic status as a third variable, as lower socioeconomic status can predict both PG and lower scores on openness (92, 93).

H5 (expecting a positive association between GP and extroversion) was not supported by the findings as the overall correlation was negative (*r* = −0.083). An a priori assumption was that a positive correlation between extroversion and GP would be in agreement with the notion of extroverts as individuals searching for social, exciting settings, which conceivably could lead to high gambling involvement. Also, extroversion is generally found to be associated with some addictions (20, 26). Whiting et al. (35) found that extroverted gamblers can be found in gambling environments where social interactions are a natural part of the gambling activity, such as poker-games or horse racing. Still, the present results did not support H5, which may be explained in several ways. Müller et al. (25) found that most gamblers are introverted, which may motivate them to search for social contact and friendships in virtual environments (94), including virtual gambling environments (25). Notably, many types of gambling (e.g., slot machines and online gambling) represent purely solitary activities, which conceivably may be more alluring for individuals with lower scores on extroversion. Lower extroversion scores have been found to be associated with social anxiety (95). For gamblers who feel socially anxious or uncomfortable, solitary gambling activities may help them to focus exclusively on the gambling activity, without increased stress levels due to uncertainty in social settings. Nevertheless, further studies should examine the association between extroversion and different types of gambling in more details.

We assumed that several moderators could impact the strength of correlations between the five personality traits and PG. Accordingly, meta-regressions were conducted in light of the large between-study variances detected. However, no significant findings were detected for neuroticism, conscientiousness, agreeableness and openness. Thus, the heterogeneity in findings on these four personality traits remains unexplained. The meta-regression did, however, show that diagnosis was significantly associated with the between-study variance for the meta-analysis of extroversion, suggesting more negative effect sizes for samples with participants with a formal gambling diagnosis than for samples without. One explanation to this finding is that alcohol abuse, anxiety, and depression have been reported to be highly prevalent among problematic gamblers (96) and represent disorders often related to social alienation and withdrawal; which, as such, may explain the meta-regression results for extroversion.

Overall, heterogeneity remained by and large unexplained. This implies that future studies and meta-analyses should consider the assessment of other, and possibly more relevant moderators (such as type of games, e.g., lottery, poker games, horse racing, internet gambling, and socioeconomic factors such as age and gender), in order to be able to explain larger proportions of the between-study variances.

### Strengths and Limitations of the Included Studies

The studies included in the present meta-analysis originated from a variety of countries. Still, few studies were conducted in non-Western and developing countries. Furthermore, the studies showed a broad variation in terms of study quality, as shown by the Newcastle-Ottawa assessment in Table 2. For instance, Roy et al. (86) and Barnes and Parwani (71) received rather low scores, which may reflect that those studies were relatively old and thus, not fulfill common criteria of scientific scrutiny of today. Other studies consisted of relatively small sample sizes with a specific group in focus. The studies of Chiu and Storm (31), MacLaren et al. (78), and McGrath et al. (33), for instance, consisted of students, while Müller et al. (25), Myrseth et al. (21), and Mann et al. (80) included problem gamblers seeking treatment. In contrast, there were only one cross-sectional study consisting of a large sample of more than 9,000 randomly selected participants (22). Hence, further studies should comprise large samples of randomly selected participants. Another major limitation of the identified literature is that very few studies (30, 75, 87) used longitudinal designs. We did, therefore, not conduct a meta-analysis of such data. Still, longitudinal data on the relationship between PG and personality are of vital importance in order to elucidate directionality and potential causality between PG and personality.

### Strengths and Limitations of the Present Meta-Analysis

The present meta-analysis has several strengths. It was conducted in line with the PRISMA guidelines (36). Searches were conducted across several databases, including search in gray literature, although the gray literature yielded no unique hits. To ensure reliability regarding quality assessment and effect size data, the included studies were coded independently by two authors where disagreements were resolved by discussion or by consulting a third team member. Records showed overall good initial agreement between the raters, suggesting high interrater reliability. Authors were contacted when relevant data were missing.

The main limitation of the present meta-analysis is that definitions of PG were not the same across studies. It cannot be ruled out that the associations between PG and personality may vary as a function of the various operationalizations of both PG and personality. The large number of operationalizations did, however, preclude meaningful subgroup analysis of results from specific operationalizations. In addition, there were few studies comprising randomized samples. In addition, the target population (problems gamblers), consisted of various types of gamblers, reflecting large heterogeneity in terms of types/groups of gamblers. This implies restrictions in terms of external validity. Also, limited generalizability relates to the broad usage of personality measures and the large range of gamblers included (from students to pathological gamblers). Another limitation pertains to the screening process of relevant studies, which initially was based on title and abstract. Hence, it cannot be ruled out that a few relevant studies may have been excluded, although relevant information usually is presented in the title or abstract. A final limitation is that the funnel plots showed asymmetry in four out of five meta-analyses, suggesting publication bias. The trim and fill procedure did however aim to correct for this. The discrepancies between the original and adjusted effect sizes were small to moderate, suggesting that the data and conclusions were not influenced by publication bias.

### Clinical Implications

The positive association between PG and neuroticism should alert clinicians to the vulnerability of those suffering from PG regarding experiencing dysphoric states. Therapists should therefore consider to address this vulnerability specifically. In this context clinicians should also be aware that many gamblers gamble in order to escape dysphoric emotional states (97). Neuroticism has also been associated with treatment relapse (23), hence relapse prevention should be given extra thought when treating patients with high scores on neuroticism. Conscientiousness was inversely related to PG. Hence many patients have low scores on this trait. Clinicians may therefore make an extra effort to motivate these patients in terms of compliance (e.g., with home tasks) as this has been shown to be lowered in those with low scores on conscientiousness (98) and also include exercises helping patients with long-term goal planning. Low scores on conscientiousness has also been associated with treatment relapse (23), suggesting that increased focus on relapse prevention might be called for. Agreeableness was inversely related to PG. Therapists should be aware that forming a good therapeutic alliance with those having low scores on agreeableness is more difficult than with those with higher scores (98). Those with low scores on this trait often cope with interpersonal difficulties in destructive ways (99), which may be addressed specifically in therapy. Clinicians should also be aware that those with low scores on agreeableness seem to drop out of treatment more often than those with higher scores (23). Also, openness was inversely related to PG. Those with low scores on this trait may be less receptive to the idea of psychotherapy itself. In therapy they may however be particularly responsive to concrete and practical suggestions (100). Extroversion showed the weakest association with PG. This trait has been found to be related to gambling motives. Those with high scores on extroversion seem to be incentivized to gamble due to social motives (101). In a similar vein, an inverse association between online gambling and extroversion has been found (102). Hence, clinicians may consider gambling motives in relation to extroversion in therapy. Overall, several of the traits in the five-factor model of personality are related to several therapeutic challenges which clinicians should be aware and take into account when treating patients with PG.

## Conclusions

The present meta-analysis integrated and synthesized previously findings on the association between PG and the personality traits of the five-factor model, showing significant correlations between all the five personality traits and PG. The postulated hypotheses were supported for four of the traits, albeit not for extroversion. Only one of the moderators (diagnosis) turned out significant for one personality dimension (extroversion), suggesting the need to identify relevant moderators in future research. The results have implications for understanding of vulnerabilities related to development of gambling problems and shed important light on issues related to prevention and treatment (e.g., such as compliance and drop-out).

## Data Availability Statement

The meta-analysis datasets for this review study can be provided by contacting the corresponding author.

## Author Contributions

SP assisted by RS, KB, AR: conceptualization. RS, KB, and AR: literature search and study coding. RS, KB, and AR assisted by SP: data analysis. All authors drafting and critically revising the manuscript, finalizing the paper and approval of submitted version.

## Funding

This study was funded by the Norwegian Competence Center for Gambling and Gaming Research, University of Bergen, Norway.

## Conflict of Interest

The authors declare that the research was conducted in the absence of any commercial or financial relationships that could be construed as a potential conflict of interest.

## Publisher's Note

All claims expressed in this article are solely those of the authors and do not necessarily represent those of their affiliated organizations, or those of the publisher, the editors and the reviewers. Any product that may be evaluated in this article, or claim that may be made by its manufacturer, is not guaranteed or endorsed by the publisher.
