# Supplementary figures and images for: The Relationship Between Gambling Problems and the Five-Factor Model of Personality: A Systematic Review and Meta-Analysis
Source: Front Psychiatry. 2021 Oct 12;12:740235. doi: 10.3389/fpsyt.2021.740235 (PMC8545825; doi:10.3389/fpsyt.2021.740235)

*Neuroticism*


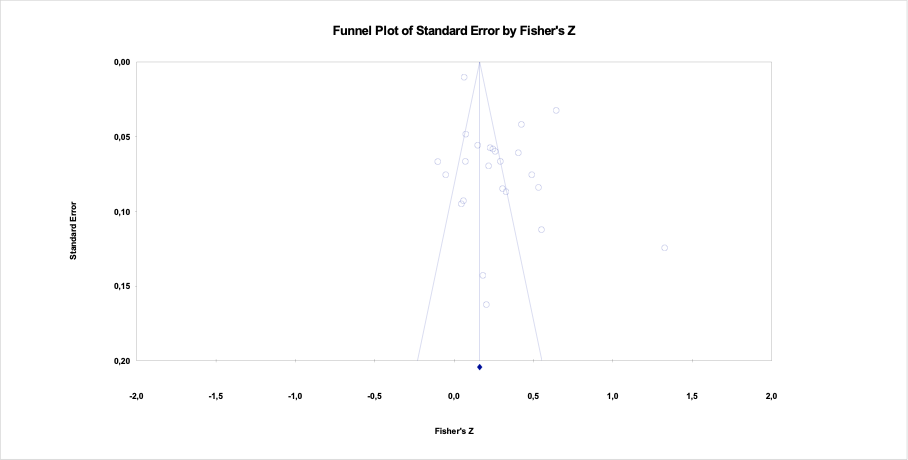


*Conscientiousness*


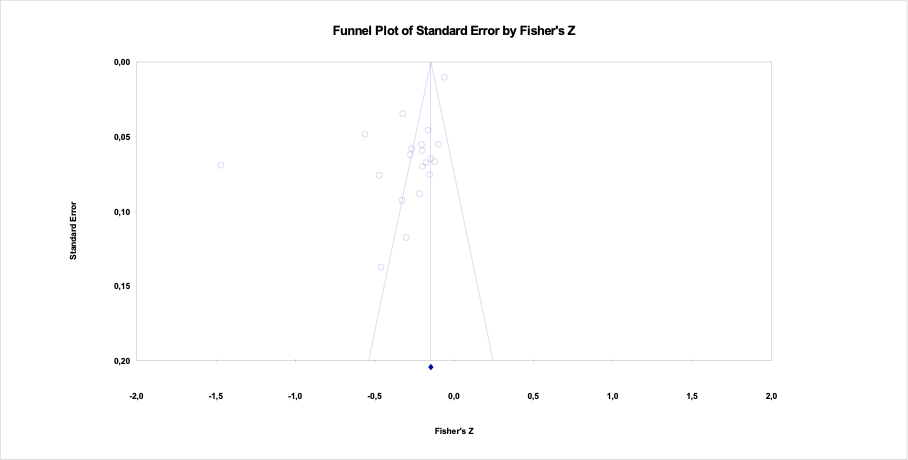


*Agreeableness*


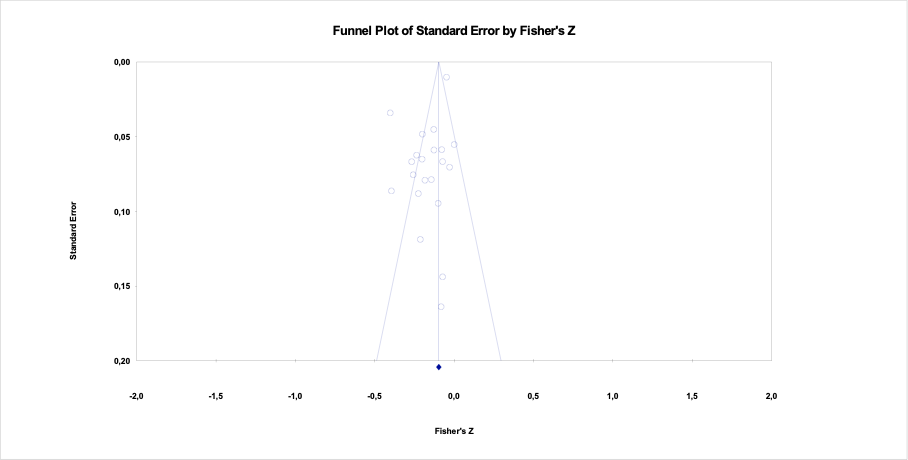


*Openness*


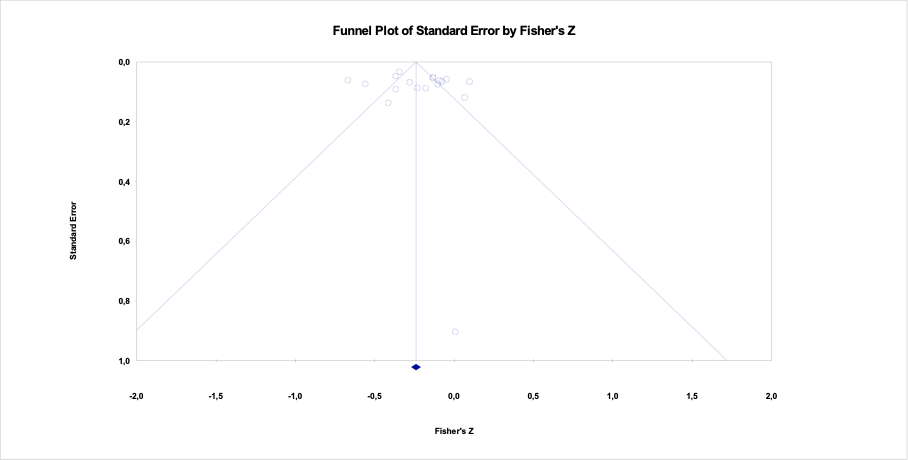


*Extroversion*


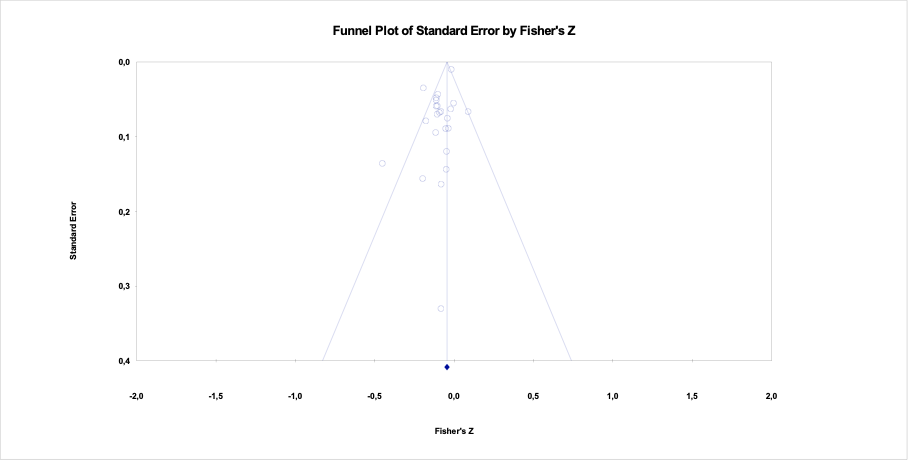

Supplement: Supplementary file 1 [file Data_Sheet_1.docx]
